# Breathing: old and fresh breezes in Orthodontics

**DOI:** 10.1590/2177-6709.25.5.007-008.edt

**Published:** 2020

**Authors:** Flavia Artese

**Affiliations:** 1 Universidade do Estado do Rio de Janeiro, Departamento de Odontologia Preventiva e Comunitária (Rio de Janeiro/RJ, Brazil).

Upon concluding a lecture on dentoalveolar bimaxillary protrusions and their treatment possibilities, I was asked many questions about the association between the reduction in the volume of dental arches after extractions and the development of respiratory disorders, notably obstructive sleep apnea (OSA). I admit that at that time I was not aware of the current evidence on the correlation between these two factors, which led me to question whether Orthodontics was causing OSA by retracting anterior teeth for almost a century. Although I searched for the answers to those questions, another one did not leave my mind. In that lecture, among the options for treating bimaxillary protrusions, the full distalization of dental arches using skeletal anchorage was also presented. And, although the dental arches are retracted, its possible association with OSA was not discussed in the question session. Would there be differences between these treatment possibilities with regard to the final position of the incisors in the anteroposterior direction?

The final results of bimaxillary protrusion cases published in the Special Topic of this issue of the DPJO (page 66) by Dr. Henrique Vilella, treated without extractions, and with arch distalization anchored on intra or extra-alveolar mini-implants, are very similar to cases treated with premolar extractions. This suggests that, in spite of effectively retracting the anterior teeth with both possibilities of treatment, we still carry biases with regard to tooth extractions. Are they the only cause in reducing the volume of dental arches, and not full arch distalization? And to what extent are changes of the dentoalveolar region in the sagittal direction associated with the effects on the airways?

The fact is that this association still seems to be unclear. There are few studies evaluating changes in the anteroposterior position of the incisors and their impact on breathing,^1^ with inconclusive results due to heterogeneous samples and substitutive outcomes, such as airway volume instead of apnea and hypopnea indexes (AHI) and oxygen saturation. In fact, changes in basal bone, such as those obtained from surgical maxillomandibular advancements, seem to have more robust evidence on their positive impact on the treatment of OSA, than just tooth movements in the sagittal direction.^2^

Some aspects of OSA were already described in the 19th century as Pickwick’s Syndrome, in reference to the little fat boy, Joe, in Charles Dickens’s novel “The Pickwick Papers”. However, it was in a 1981 Lancet paper that Sullivan and collaborators published the first use of continuous positive airway pressure (CPAP) as a way of treating OSA.^3^ Since then, this condition has been approached and integrated with several specialties in the health field, as it is a multifactorial problem and often requires multidisciplinary treatment. It is known that in patients with high indexes of apnea and hypopnea, the gold standard treatment is the use of CPAP, although adherence to this approach is low. In this respect, Orthodontics can help reduce the severity of AHI with mandibular advancement devices. There are several models and with different characteristics, such as the DIORS device presented in the article by Drs. Barbosa and collaborators in this issue (page 44). Its conclusion is quite pertinent and agrees with the results of the systematic review that establishes that the use of mandibular advancement devices will not reach CPAP results, but serve instead as an alternative treatment, even with smaller results in AHI in patients resistant to CPAP.^4^

Despite the importance given to Orthodontics in recent decades in its relationship with OSA, whether in terms of its treatment or prevention through our therapeutic choices, which seems very new, has indeed been available for a long time by the hands of Andrew Haas. I had the unique opportunity to listen to him at one of the Angle Society meetings, where he told how the idea of ​​rapid maxillary expansion (RME) also arose from the interest of otolaryngologists with the intention of changing the internal anatomy of the nose. His classic papers^5^ ^,^ ^6^ report an increase in the volume of the nasal cavity after RME, resulting in the clinical improvement of mouth-breathing patients.

In this issue of DPJO, Dr Bruno and collaborators evaluated the changes in the morphology of the nasal septum after RME, and did not observe changes neither in the volume nor in the deviation of the septum (page 51). However, although we cannot change the anatomical aspects of the nasal septum, we can be the first specialists to diagnose the consequences of oral breathing in children, such as constricted upper arches, posterior crossbites and high palates. Thus, we have a huge responsibility to explore even further this diagnosis, either through a specific questionnaire for the child’s respiratory evaluation, or by a referral to an otolaryngologist.

If we still do not know whether dental changes in the sagittal direction have an influence on breathing, for transverse expansions the evidence is very clear. A systematic review published by Camacho et al.^7^ shows that RME consistently improves both AHI and minimum oxygen saturation in the short-term, that is, during a follow-up of 3 years.

 The search for fresh breezes in Orthodontics has been within our reach since the 60s. In fact, what we have new is the diagnostic approach towards breathing, since using a routine procedure, such as RME, we are able to correct a very serious problem. Even more important is to know the consequences of OSA and share our diagnosis with a multidisciplinary group, where the scope of our specialty goes beyond occlusion and aesthetics, ensuring basic functions for a healthy life of our patients.

Good reading!

## References

[1] Ng JH, Song YL, Yap AUJ (2019). Effects of bicuspid extractions and incisor retraction on upper airway of Asian adults and late adolescents A systematic review. J Oral Rehabil.

[2] Camacho M, Noller MW, Del Do M, Wei JM, Gouveia CJ, Zaghi S, Boyd SB, Guilleminault C (2019). Long-term results for maxillomandibular advancement to treat obstructive sleep apnea A meta-analysis. Otolaryngol Head Neck Surg.

[3] Sullivan CE, Berthon-Jones M, Issa FG, l Eves (1981). Reversal of obstructive sleep apnoea by continuous positive airway pressure applied through the nares. Lancet.

[4] Schwartz M, Acosta L, Hung YL, Padilla M, Enciso R (2018). Effects of CPAP and mandibular advancement device treatment in obstructive sleep apnea patients a systematic review and meta-analysis. Sleep Breath.

[5] Haas AJ (1970). Palatal expansion just the beginning of dentofacial orthopedics. Am J Orthod.

[6] Haas A (1961). Rapid expansion of the maxillary dental arch and nasal cavity by opening the midpalatal suture. Angle Orthod,.

[7] Camacho M, Chang ET, Song SA, Abdullatif J, Zaghi S, Pirelli P, Certal V, Guilleminault C (2017). Rapid maxillary expansion for pediatric obstructive sleep apnea A systematic review and meta-analysis. Laryngoscope.

